# Central retinal artery occlusion after intravitreal brolucizumab injection for treatment-naïve neovascular age-related macular degeneration; a case report

**DOI:** 10.1186/s12886-024-03452-3

**Published:** 2024-04-29

**Authors:** Sung Hwa Hong, Hoon Dong Kim

**Affiliations:** Department of Ophthalmology, College of Medicine, Soonchunhyang University, Cheonan, Republic of Korea

**Keywords:** Age-related macular degeneration, Brolucizumab, Central retinal artery occlusion, Intraocular inflammation

## Abstract

**Background:**

To report a case of central retinal artery occlusion (CRAO) after intravitreal injection of brolucizumab for a treatment-naïve neovascular age-related macular degeneration (nAMD) patient without comorbid cardiovascular disease history.

**Case presentation:**

A 79-year-old Asian male without a cardiovascular disease history such as diabetes or hypertension underwent three times of monthly consecutive intravitreal brolucizumab injections for treatment of progressed nAMD in his left eye. Two days after the third injection, the patient presented with acute painless visual loss. Typical retinal whitening with a cherry red spot was observed on the fundus photograph, and retinal swelling with hyper-reflectivity was also identified on the optical coherence tomography (OCT) scan. On the fundus fluorescein angiography, arm-to-retina time and arteriovenous transit time were remarkedly delayed, but clinical findings suggesting an intraocular inflammation (IOI) were not observed. Therefore, CRAO was diagnosed, and anterior chamber paracentesis was administrated immediately. However, there had been no improvement in visual acuity during the follow-up period of three months, despite prolonged oral steroid and anti-platelet agent medication.

**Conclusions:**

In rare cases, patients without cardiovascular comorbidities can develop CRAO after intravitreal brolucizumab injection without gross evidence of IOI. Therefore, CRAO should always be in consideration and careful observation is required after intravitreal brolucizumab injection for nAMD patients with old age, even if the patient does not have any other cardiovascular disease history.

## Background

Anti-vascular endothelial growth factor (VEGF) therapy has been known as a gold standard treatment modality for neovascular age-related macular degeneration (nAMD). Intravitreal anti-VEGF injections including bevacizumab, ranibizumab, and aflibercept have been used widely for nAMD patients. Brolucizumab (Beovu®; Novartis Pharma AG, Basel, Switzerland) is an anti-VEGF agent composed of variable light and heavy chain domains of humanized monoclonal antibodies that bind to the isoforms of VEGF-A, and it was approved for the treatment of nAMD in 2019 [1, 2]. With a small molecular weight of approximately 26 kDa, it demonstrates excellent tissue penetration and higher molar concentration [1, 2]. Higher tissue penetration allows it to reach the choroid and retinal pigment epithelium (RPE) more easily [3]. Compared to previous anti-VEGF agents, smaller molecular weight enables injection of a relatively higher molar concentration for equivalent volume into the vitreous body, expected to reduce the burden of frequent injections and the risk of complications on patients by prolonging the injection interval [1, 2].

However, substantial safety issues emerged as serious adverse effects were reported following brolucizumab injection. In particular, intraocular inflammation (IOI) including retinal vasculitis with or without retinal artery occlusion (RAO) has been identified as the main cause of these concerns associated with brolucizumab. According to the results of HAWK and HARRIER studies, the incidence of IOI was higher in the brolucizumab (6 mg) injection group, compared to the aflibercept injection group [4]. The major clinical manifestations of documented IOIs were iritis and uveitis, which were reduced after topical or periocular steroid treatments [4]. However, some cases such as RAO accompanied by retinal vasculitis showed relatively poor visual prognosis. Given its unfavorable impact on visual prognosis, RAO demands more attention than other adverse events. Brolucizumab injection-related RAO reported to date mainly occurred with IOI, and most patients had underlying cardiovascular disease [4].

In this report, we present a case of central retinal artery occlusion (CRAO) that occurred alone without gross signs of IOI after consecutive three brolucizumab injections as a loading dose in a nAMD patient who does not have any other comorbid cardiovascular disease.

## Case presentation

**Fig. 1:**
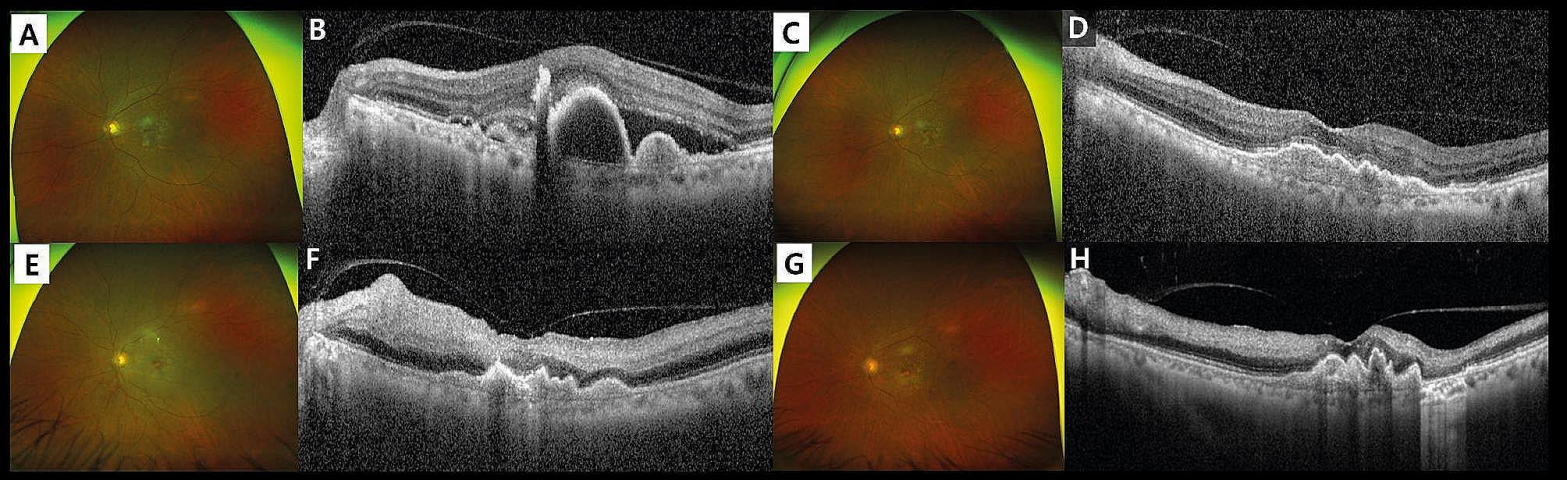
Consecutive changes of fundus photographs and optical coherence tomography (OCT) scans in the left eye of a 79-year-old male who received three times of monthly intravitreal brolucizumab injections. Newly developed choroidal neovascularization (CNV) with subretinal fluid (SRF) was identified (**A**, **B**). After the second injection of intravitreal brolucizumab, CNV and SRF was resolved on the OCT scan (**D**), and there were no posterior intraocular inflammation (IOI) findings (**C**). Fundus photograph two days after the third injection showed an retinal whitening with a cherry red spot without significant inflammatory signs (**E**), and an OCT scan revealed hyper-reflectivity in the inner retina with retinal swelling (**F**). One month after his last injection, no evidence of posterior IOI was seen (**G**), and OCT revealed the progression of atrophic inner retinal thinning induced by ischemia (**H**)

**Fig. 2:**
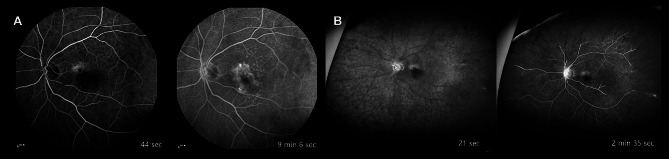
Changes of fundus fluorescein angiography (FFA) findings in the patient’s left eye. Hyperfluorescence due to leakage from CNV was identified before the first intravitreal brolucizumab injection (**A**), and retinal vascular filling of the dye was observed normally (A). Two days after the third intravitreal brolucizumab injection, the wide-field FFA images revealed remarkable delays in arm-to-retinal time and arteriovenous transit time (B). Posterior segment IOI signs including perivascular leakage or staining were not found in wide-field FFA (**B**)

A 79-year-old Asian male under regular follow-up for two years with non-neovascular AMD in both eyes complained of blurred vision in the left eye. He said the blurring symptom started two weeks ago. He had no documented medical history including hypertension, diabetes, and other cardiovascular diseases. In addition, intravitreal injection has not been performed previously. His best corrected visual acuity (BCVA) in the left eye decreased from 20/30 to 20/60, compared to the last visit. Fundus photograph in the left eye showed multiple drusen and newly developed choroidal neovascularization (CNV) (Fig. 1, A). Optical coherence tomography (OCT) scan image also revealed CNV with subretinal fluid (SRF) and drusenoid pigment epithelial detachment (PED) (Fig. 1, B). On fundus fluorescein angiography (FFA), there was hyperfluorescence by leakage due to CNV in the macular area of the left eye (Fig. 2, A).

Diagnosed as having progressed to nAMD, three times of intravitreal monthly injections of brolucizumab (6 mg, 0.05 mL of 120 mg/mL solution) were administered to the left eye as an initial loading dose therapy for nAMD. He responded to the first and second monthly injections with regression of CNV and resolution of SRF (Fig. 1, C, D), and his BCVA was slightly improved to 20/50. In addition, no significant subjection symptoms of the patient were noticed until the second injection.

The patient presented with acute painless visual loss in the left eye two days after the third intravitreal brolucizumab injection. His BCVA had deteriorated to counting fingers at 10 cm with a relative afferent pupillary defect in the left eye. There were no inflammatory signs in the anterior chamber. Fundus photographs showed retinal whitening on the posterior pole with a macular cherry-red spot sign (Fig. 1, E). The cotton wool patches were also found along the superior major arcade area. The OCT scan image demonstrated generalized retinal swelling with hyperreflectivity in the inner retina (Fig. 1, F). Furthermore, the wide-field FFA revealed markedly delayed arm-to-retina time and arteriovenous transit time in the left eye (Fig. 2, B). There was no evidence suggesting IOI including anterior chamber inflammation, vitreous opacity, retinal vascular sheathing, and retinal infiltrations. Perivascular leakage or staining which suggests a retinal vasculitis was not identified in the FFA images, either (Fig. 2, B).

With the diagnosis of CRAO, anterior chamber paracentesis was conducted immediately, and topical intraocular pressure lowering agent, oral steroid, and oral anti-platelet agent were administered for 1 month. No further intravitreal anti-VEGF injection was performed. One month after his last injection, his BCVA was maintained as counting fingers. No evidence of posterior IOI was still observed, and the progression of atrophic inner retinal thinning induced by ischemia was also noticed on the OCT scan (Fig. 1, G, H). Further evaluations about a possibility of cardiovascular embolism including cardiac or carotid neck ultrasounds could not be performed, because he refused additional workup for the eyes.

There had been no improvement in visual acuity during the follow-up period of three months despite prolonged oral steroid and anti-platelet agent medication. There was no recurrence of nAMD for three months after the cease of intravitreal brolucizumab injection. A generalized atrophic retina with sclerotic vessels was found on the fundus photograph in the left eye, and inner retinal thinning caused by ischemia was more progressed on the OCT scan three months after the occurrence of CRAO.

## Discussion and conclusions

Brolucizumab is a single-chain antibody fragment binding to VEGF-A and blocking the activation of angiogenic signal transduction [5]. Being an anti-VEGF agent with a smaller molecular weight, compared to other previously approved VEGF antagonists for nAMD, there was anticipation that brolucizumab would hold an advantage over its counterparts. In a preclinical study on non-human primates, brolucizumab showed favorable tissue penetration rates in the retina and RPE/choroid at 42% and 18%, respectively, compared to intravitreal concentrations after vitreous injection [3]. Due to its superior solubility and stability, it was expected to be able to deliver a higher concentration compared to other anti-VEGF agents, thereby enabling prolongation of the injection interval and the potential to alleviate the burden with less frequent injections [1, 2]. According to HAWK and HARRIER clinical studies for 96 weeks, the brolucizumab injection group showed non-inferiority on BCVA and better anatomical outcomes than the aflibercept injection group [4].

However, these initial expectations were overshadowed by concerns, as a strong correlation emerged between brolucizumab and IOI, including a few cases of retinal vasculitis with or without RAO. The occurrence of IOI following the administration of a conventional anti-VEGF agent has been reported [6]. In the HAWK and HARRIER studies, however, the brolucizumab injection group had a significantly higher incidence of IOI at 4.7%, compared to the aflibercept group which had a much lower rate of 0.6% [4]. Similarly, Saba et al. reported that the incidence of IOI was comparable at 4.8% in the 8-month post-marketing surveillance analysis [7].

Most of the reported IOIs were iritis and uveitis, which could subside spontaneously or be reduced after topical or periocular steroid treatments. Visual prognosis of most patients who underwent IOI revealed relatively good during the follow-up period [4]. However, there were several cases with relatively poor visual prognosis, such as retinal vasculitis with RAO [4, 7–9]. A retrospective analysis of 26 eyes from 25 patients evaluated retinal vasculitis following the administration of brolucizumab, and 92% of cases of retinal vasculitis were accompanied by anterior chamber inflammation and/or posterior segment inflammation [8]. Among them, occlusive vasculitis was reported in 85% of the eyes. Most cases of retinal vasculitis showed improvement with steroid treatment, but some cases with RAO exhibited limited response even with steroid treatment [8].

Ocular arterial thromboembolic events reported in HAWK and HARRIER studies occurred in a total of 10 cases in the brolucizumab 6 mg injection group, and seven of which occurred concurrently with IOI, compared to 3 cases in the brolucizumab 3 mg injection group [4]. Patients in all IOI cases had cardiovascular risk diseases such as hypertension and cardiac arrhythmias as underlying diseases [4]. In a previous report, Sentaro et al. suggested a possibility that retinal vascular occlusion after brolucizumab injection could occur without vasculitis and IOI [9]. As it is well known that VEGF itself plays an important role in maintaining vascular endothelial cell integrity, it is thought that the VEGF inhibitor itself may interfere with the physiologic function of the vessel and cause occlusion [10, 11]. The mechanism explaining RAO after anti-VEGF injection still has not been fully elucidated. It has been proposed that the relatively potent and long-lasting VEGF inhibitory effect of brolucizumab may lead to the damage of retinal vascular endothelial cells, consequently resulting in retinal vascular occlusion [9]. Unfortunately, a possibility of the association with cardiovascular embolism risk has not been evaluated in this case, because the patient did not want an additional assessment.

In this case, the patient presented typical CRAO findings two days after administering the third monthly injection of intravitreal brolucizumab. Unlike other RAO patients previously reported after injection of brolucizumab, the patient had no underlying cardiovascular disease such as hypertension, diabetes, or dyslipidemia. In addition, there was no gross evidence of IOI in the comprehensive ophthalmologic examinations performed before or after the occurrence of CRAO, and there was no improvement even with a steroid treatment. It is thought that there is a possibility of occlusive vasculitis due to the presence of microscopic or minimal inflammatory signs, which could be detected under retinal imaging tools such as fundus photography or FFA.

Although the incidence can be rare, this case shows the possibility of retinal vascular occlusive events without definite evidence of IOI in eyes without cardiovascular risk factors for RAO. Since the visual prognosis after the occurrence of CRAO is poor, the possibility of CRAO after intravitreal brolucizumab treatment should always be in consideration and careful observation is required during the short-term follow-up period after the intravitreal brolucizumab injection for nAMD patients with old age, even if the patient does not have any other comorbid cardiovascular disease history.

## Data Availability

All data generated or analyzed during this study are included in this published article. Further inquiries can be directed to the corresponding author.

## Abbreviations

CRAO: Central retinal artery occlusion
nAMD: neovascular Age-related macular degeneration
OCT: Optical coherence tomography
IOI: Intraocular inflammation
VEGF: Vascular endothelial growth factor
RPE: Retinal pigment epithelium
RAO: Retinal artery occlusion
BCVA: Best corrected visual acuity
CNV: Choroidal neovascularization
SRF: choroidal neovascularization
PED: pigment epithelial detachment
FFA: pigment epithelial detachment
